# Outcomes of Septal Myectomy beyond 65 Years, with and without Concomitant Procedures ^†^

**DOI:** 10.3390/jcm10163499

**Published:** 2021-08-08

**Authors:** Robert Pruna-Guillen, Daniel Pereda, Manuel Castellà, Elena Sandoval, Alessandro Affronti, Ana García-Álvarez, Juan Perdomo, Cristina Ibáñez, Paloma Jordà, Susanna Prat-González, Jorge Alcocer, Clemente Barriuso, Jaume Llopis, Eduard Quintana

**Affiliations:** 1 Department of Cardiovascular Surgery, Institut Clínic Cardiovascular, Hospital Clínic de Barcelona, University of Barcelona Medical School, 08036 Barcelona, Spain; pruna@clinic.cat (R.P.-G.); dpereda@clinic.cat (D.P.); mcaste@clinic.cat (M.C.); esandova@clinic.cat (E.S.); affronti@clinic.cat (A.A.); alcocer@clinic.cat (J.A.); barriuso@clinic.cat (C.B.); 2 Department of Cardiology, Institut Clínic Cardiovascular, Hospital Clínic de Barcelona, University of Barcelona Medical School, 08036 Barcelona, Spain; anagarci@clinic.cat (A.G.-Á.); pjordab@clinic.cat (P.J.); suprat@clinic.cat (S.P.-G.); 3 Anesthesiology Department, Hospital Clínic de Barcelona, 08036 Barcelona, Spain; jmperdom@clinic.cat (J.P.); cribanez@clinic.cat (C.I.); 4 Department of Genetics, Microbiology and Statistics, University of Barcelona, 08036 Barcelona, Spain; jllopis@ub.edu

**Keywords:** hypertrophic obstructive cardiomyopathy, septal myectomy, mitral regurgitation, cardiomyopathy

## Abstract

Introduction and objectives: Septal myectomy remains the first septal reduction therapy for hypertrophic obstructive cardiomyopathy in young patients and those requiring concomitant procedures. Its role in advanced ages is questioned due to perceived increased risk. We assess the outcomes of surgical relief of obstruction in patients beyond 65 years old. Methods: A single-center retrospective review of patients ≥ 65 years old undergoing septal myectomy through median sternotomy between April 2015 and February 2020. Results: We identified 52 patients. Mean age was 71.8 ± 4.9 years; 36 (69.2%) were females. All were symptomatic. Mean highest LVOT gradient was 90 ± 39 mmHg. All patients had systolic anterior motion (SAM) of the mitral valve and 36 (69.2%) ≥ moderate mitral regurgitation. Additional LVOT interventions beyond myectomy were performed in 34 (65.4%). At least one other cardiac concomitant procedure was performed 44 (84.6%). No perioperative mortality in elective surgery occurred. One patient (1.9%) developed atrio-ventricular block. Postoperative mean gradient was 4.3 ± 1.9 mmHg, with 46 (88.4%) achieving complete resolution of obstruction. Mitral regurgitation was reduced to grade ≤ I in 46 (88.5%). Mean follow-up time was 2.3 ± 1.2 years and 82% of patients were in NYHA I. Survival at 2 years was 98%. Conclusion: Septal myectomy in the elderly is a safe and effective operation despite the need for concomitant procedures. LVOT interventions beyond septal myectomy to relieve obstruction are common in this advanced cohort of hypertrophic cardiomyopathy patients. This operation carried at experienced centers seems an unmatched therapeutic option.

## 1. Introduction

Hypertrophic cardiomyopathy (HCM) is the most prevalent inherited cardiomyopathy. A disproportionate ventricular wall thickness (≥15 mm) in the absence of other diseases accountable for such hypertrophy establishes the diagnosis. HCM is a diagnosis of exclusion, often challenging due to different phenotypic expressions [1] presenting at any point in a lifetime with variable clinical repercussions. In certain patients with clinically significant obstruction, it is difficult to differentiate an inherited myocardial disease from secondary ventricular hypertrophy [2]. Approximately 40% of HCM probands have a nonfamilial subtype with later onset and less severe clinical course. Patients with clinically diagnosed HCM after the age of 65 may be more likely to remain genetically elusive [3]. 

Left ventricular outflow tract (LVOT) obstruction is defined when a gradient ≥ 30 mmHg is demonstrated, which has a detrimental effect on survival [4]. In patients with obstruction (≥50 mmHg) and severe drug-refractory symptoms, invasive therapy is recommended [5]. Dynamic obstruction arises from an interplay between septal hypertrophy and other structural LVOT abnormalities causing decreased forward blood flow and increased left-sided filling pressures. Additionally, as a result of such abnormal physiology, variable degrees of mitral regurgitation follow systolic anterior motion (SAM) of the mitral valve. The septal hypertrophic component of the disease can be addressed by septal myectomy (SM) and occasionally by alcohol septal ablation (ASA). The proof of a genetic abnormality leading to HCM has not been a requirement to justify an intervention when a symptomatic and refractory obstruction is present.

Septal myectomy remains the first septal reduction therapeutic option according to recent AHA/ACC guidelines [5]; however, in Europe, ASA has eclipsed SM surgery [6] despite the increased risk of complete atrioventricular blockage, residual obstruction and need for reintervention [7,8,9,10,11].

The role of SM at advanced ages is questioned based on perceived increased perioperative risk [12]. This subgroup of patients older than 65 years, constitutes an important segment of the disease that has been poorly defined and is seldomly reported [13,14]. The objective of our study is to review the profile and outcomes of SM for patients older than 65 years with and without concomitant procedures.

## 2. Materials and Methods

The Research Ethics Committee approved this study and waived the need for individual consent (IRB HCB/2019/0676) in July 2019.

### 2.1. Study Population

The study constitutes a single-center retrospective review of patients older than 65 years who underwent SM through median sternotomy between April 2015 and February 2020. Only patients with documented dynamic LVOT obstruction (gradient ≥ 50 mmHg) were included. Patients undergoing concomitant procedures were also included, except for mitral valve replacements. We selected the chronological cut-off age of 65 to focus on a cohort where the availability of literature to guide practice and informed-decision making is deficient. This threshold is the definition of elderly by the World Health Organization. Data collection was closed in August 2020 when all patients had a minimum of 6 months of postoperative follow-up.

During the same study period 4 additional patients older than 65 years qualified for SRT. Two underwent isolated ASA. The indication to pursue ASA was perceived high surgical risk and/or patient-heart team preference (one was an 82-year-old with prior chest radiation). The other 2 patients ultimately received optimal medical treatment and did not undergo any form of SRT (1 due to patient’s reluctance to invasive therapies and the other was an 85-year-old that was deemed too frail to undergo any form of SRT). Both remain alive at the time of this report.

### 2.2. Exclusion of HCM Patients Receiving a Mitral Valve Replacement

During this period analysis, two HOCM patients with mitral valve endocarditis received a mitral valve replacement. Two additional HOCM patients underwent planned mitral and aortic valve replacement due to severe degenerative multivalve disease.

One HOCM patient underwent mitral valve replacement without myectomy after intraoperative assessment due to the presence of abnormal papillary muscle attached to the body of the anterior leaflet and severe fibrocalcific mitral apparatus precluding a mitral sparing approach.

Three additional patients were scheduled for extended myectomy and mitral intervention. In two of them, prolapse and leaflet restriction were present preoperatively; unacceptable residual mitral regurgitation led to mitral replacement after myectomy and mitral intervention. In one patient with prominent mitral leaflets and non-severe LV hypertrophy, a myectomy and edge-to-edge repair were attempted leading to mitral stenosis and finally mitral replacement. There was no postoperative mortality or complications in this subgroup of excluded patients.

For multiple reasons, none of these patients could have been a candidate to ASA and percutaneous mitral intervention (edge-to-edge repair) and constitute a different profile of patients beyond the current analysis.

### 2.3. Myectomy and LVOT Obstruction Relief Procedures

A transverse aortotomy and retraction of the right coronary leaflet with a malleable spatula provided exposure to the left ventricular cavity. For extended septal myectomies, the initial incision started at least 3 mm below the right coronary cusp nadir and extended leftwards towards the left trigone. In a more distal LVOT aspect resection was also continued rightwards based on individual anatomy. If sufficient resection could not be achieved from the aortic root, myectomy through the left ventricular apex was performed [15]. A residual septal thickness of at least 1 cm was pursued. Any abnormal fibrotic (chordal) or muscular attachment from the mitral leaflets or its subvalvular apparatus to the ventricular wall was divided. Assessment of secondary abnormal chordae contributing to systolic anterior motion of the mitral valve was performed as described by Ferrazzi et al. [16]. Apicoseptal muscular bands were excised. When a long anterior mitral leaflet was present, contributing to systolic anterior motion (SAM) of the mitral valve, the height of the leaflet was reduced by means of horizontal plication sutures at its base [17]. If the anterior papillary muscle contributed to obstruction, repositioning was performed.

### 2.4. Intraoperative Result Assessment

Transesophageal echocardiography judged the anatomic and functional result. Patients had provoked induced premature ventricular contractions and post ventricular beat assessment of inducible gradients before decannulation of CBP (in order to elucidate Brockenbrough-Braunwald-Morrow sign). Residual obstruction was dealt with by repeat septal or mitral apparatus intervention. Inotropic challenge was reserved for anatomies amenable to further surgical maneuvers. Contrarily, patients with no obstruction in resting conditions and complex LVOT-mitral relationship were not provoked.

### 2.5. Data Acquisition and Follow Up

Data were obtained from the departmental database and electronic charts. All patients had echocardiographic studies before hospital discharge and at follow-up (including provocative maneuvers to elucidate non-resting gradients). Patients were routinely followed at our cardiovascular surgery outpatient clinic and by their referring cardiologists.

### 2.6. Statistical Analyses

Descriptive statistics for categorical variables were reported as frequency and percentage. Continuous variables were reported as mean ± standard deviation or as median (range) as appropriate. Comparisons of paired categorical variables were performed using the paired-Wilcoxon test. Comparisons of paired continuous variables were performed using a two-sided paired *t-*test. Two-sided *p* values < 0.05 were considered statistically significant. The Kaplan–Meier method was used for survival analysis. Statistical analyses were performed using IBM SPSS Statistics for Macintosh, Version 26.0. Armonk, NY, USA: IBM Corporation.

## 3. Results

Over 4 years and 9 months, 52 patients older than 65 years underwent surgery for LVOT obstruction relief with or without concomitant procedures. The mean follow-up time was 2.3 ± 1.2 years (range 0.5–5.2 years). Data were complete for all patients and there was no follow-up loss.

### 3.1. Pre-Operative Patient Characteristics

Preoperative clinical features are summarized in Table 1. Advanced (dyspnea) heart failure—defined by New York Heart Association (NYHA) functional class III-IV—was the most frequent symptom (90.4%). One patient (1.9%) had a prior failed ASA elsewhere. Holter was available for 27 patients (52%) in the previous year. One patient (1.9%) had a recovered sudden death before surgery and 4 (7.7%) had ventricular arrhythmias on preoperative 24-h holter monitoring. Two patients (3.8%) had previously received a permanent pacemaker before the intervention and the remaining were in sinus rhythm (71.1%). Left bundle branch block was observed preoperatively in 12 (23.1%) and right bundle branch block was observed in 4 (7.7%). All patients were receiving or had attempted appropriate medical therapy. The risk of sudden death (SD) was calculated with the HCM risk-SCD tool [18].

### 3.2. Preoperative Imaging and Hemodynamic Data

Five patients (9.6%) had a left ventricle ejection fraction < 60% (lowest 50%). The mean highest intraventricular gradient was 90 ± 39 mmHg. The mean left ventricle end-diastolic diameter was 44.3 ± 7.8 mm. All patients except one had patterns of asymmetric LV hypertrophy. In 16 patients, cardiac magnetic resonance was available and 8 (50%) presented delayed myocardial enhancement compatible with fibrosis. All patients had mitral regurgitation due to SAM under provocation. Data from 37 patients that received a Swan–Ganz catheter at surgery showed a median systolic pulmonary artery pressure of 32 mmHg (range:18–130). Table 2 summarizes preoperative echocardiographic characteristics.

### 3.3. LVOT Obstruction Relief Manoeuvres, Concomitant Procedures and Intraoperative Data

The majority of operations (92.3%) were elective. However, 4 (7.7%) unstable patients could not be discharged and were operated on during the same admission. All patients underwent transaortic SM. Forty-four (84.6%) patients had an extended SM and 8 (15.4%) had a classic Morrow myectomy. Only 3 (5.8%) patients required a combined transaortic and transapical approach to complete the resection and to enlarge the LV cavity. Beyond myectomy, 34 (65.4%) patients underwent adjunct LVOT maneuvers to facilitate the resolution of obstruction and 35 (67.3%) other concomitant procedures (Table 3). To note, in 7 (13.4%) patients SM was undertaken with septal thickness ≤20 mm with addition of concomitant LVOT interventions. Symptomatic LVOT obstruction was the leading and dominant indication for sternotomy in this population. Eighteen patients (34.6%) underwent more than 2 concomitant procedures. Mean cardiopulmonary bypass time was 94 ± 34 min and aortic occlusion time was 73 ± 26 min. Two patients required a second cardiopulmonary bypass run to extend the resection, and in one also the papillary muscle was repositioned to fully relieve obstruction. No patients suffered ventricular septal defect or mitral valve injury. One aortic leaflet lesion occurred and was repaired ad hoc. A 76-year-old frail female developed a focal aortic dissection at the aortic cross-clamp site during urgent extended SM and quadruple coronary artery bypass grafting—and was replaced without clinical sequelae. Thirteen patients (25%) were extubated in the operating room and 22 (42%) did not receive any blood product transfusions during the hospital stay. 

### 3.4. Postoperative Data and Follow Up

Histological analysis of each septal specimen ruled out infiltrative HCM etiology. Table 4 shows postoperative complications and outcomes. Postoperative behavior and outcomes did not differ from isolated and concomitant groups. Six patients (11.5%) required temporary inotropic support after surgery. No perioperative mortality was observed in elective operations. A 75-year-old patient with long-standing supra-systemic pulmonary hypertension under home oxygen therapy died after urgent surgery. It was felt by the HCM team that resolution of obstruction could lead to symptomatic palliation under the auspices that she was not a candidate for other therapeutic options. Low cardiac output and septic shock lead to multiorgan failure and death.

Complete resolution of obstruction (no residual intraventricular gradient) was achieved in 46 (88.4%). In 3 (5.7%) patients—aged 74, 75 and 78—a residual gradient beyond 30 mmHg was observed (either at rest or with provocative maneuvers). Those 3 patients with residual obstruction underwent at least one concomitant procedure and obtained significant obstruction relief with an improvement of preoperative symptoms (2 became asymptomatic).

After surgery and before discharge, mitral regurgitation was mild or less in 46 (88.5%) patients. In 5 (9.6%) patients, mitral regurgitation was moderate (4 had degenerative organic mitral degeneration and 1 SAM-induced). In a 75-year-old very frail patient with a small LV, severe degenerative mitral regurgitation was accepted to avoid implantation of a mechanical valve.

In this series, 32/52 (61.5%) of patients developed left bundle branch block after SM. Despite the significant number of preoperative conduction disorders, only one patient required a permanent pacemaker during the index hospitalization. This patient was a 77-year-old that underwent aortic root/ascending replacement, SM, left atrial appendage occlusion and coronary bypass grafting.

**Figure 1:**
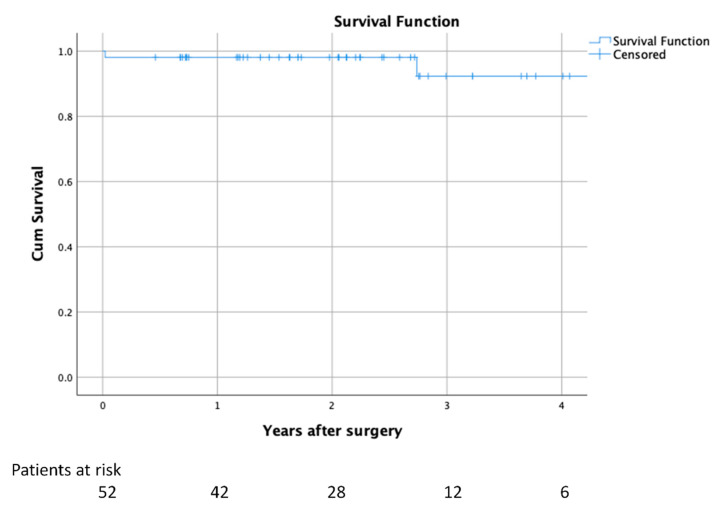
Postoperative survival.

**Figure 2:**
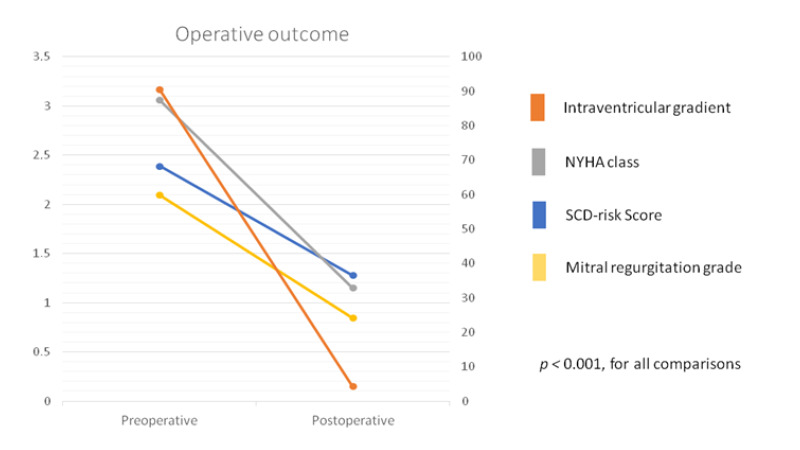
Impact of surgery.

Postoperatively, all patients experienced a significant improvement in heart failure symptoms, and all are free from angina or syncope. At follow-up 9 (19.6%) patients remain free from any beta-blocker or calcium channel blocker prescription. None of the patients have required repeated septal reduction therapy (SM or ASA) to date. One patient required a percutaneous “edge-to-edge” repair after myectomy and mitral prolapse repair. HCM risk-SCD decreased from 2.4 ± 1.7% preoperatively and to 1.3 ± 0.9% postoperatively (*p* < 0.001). There has not been any cardiac death to date. Figure 1 shows a cumulative survival of 98.1% and 92.3%, at 2 and 4 years respectively. An 81-year-old patient died 3 years after the operation as a consequence of esophageal adenocarcinoma and an 87-year-old female died 5 years after the procedure as a consequence of progressive renal failure managed with comfort measures. Figure 2 shows the change in preoperative NYHA status, LV gradient, SCD risk and mitral regurgitation (all statistically significant, *p* < 0.001) postoperatively.

## 4. Discussion

The safety profile of SM in the elderly, even with concomitant procedures, at an experienced HCM referral center is exhibited here. Despite the need for associated procedures, inclusion of urgencies and severe comorbidities, we have reproduced the outcomes of the major myectomy series with an average age of 20 years younger [19,20,21]. For this retrospective cohort, the genetic portfolio is not universally available. It is possible that some patients were not inherited HCM cases, though, this applies also to the majority of published SM series. The current analysis based on obstructive pathophysiology resolution rather than pure etiology may facilitate outcome comparison to general septal reduction therapy series. The study is subject to biases inherent to retrospective analyses with a limited number of patients.

Septal myectomy is a low-risk operation (<1%)—in specialized centers—constituting the first septal reduction option for young patients and classically for those in need of concomitant procedures. Reluctance may exist to referring elderly patients for SM under the auspices that medical therapy or ASA might be superior. Counter-intuitively, routine engagement into higher-risk operations (e.g., aorta surgery or multivalve procedures) for the elderly occurs daily at many cardiovascular practices. Unfortunately, the perception of disproportionate risk remains, leading to denial/delay of operations that hold the potential to restore quality of life. In Europe, despite higher complications with ASA (need for pacemaker, residual obstruction, repeat SRT and arrhythmia risk [7,9,22]) this alternative has overshadowed SM. Additionally, the availability of “less-invasive” options to treat concomitant cardiac diseases such as coronary stents, transcatheter valves, mitral clips, appendage occlusion devices and percutaneous ablative therapies for atrial fibrillation leads to a broader debate for the management of HCM requiring LVOT obstruction relief.

In our experience, the transformation of HOCM in a non-obstructive disease led to the resolution of heart failure symptoms for the vast majority. The absence of mortality in elective procedures, minimal need for pacemaker implantation (1.9%) and absence of bleeding/wound complications supports a liberal indication of SM in selected elderly patients. The median 1-day ICU stay for our surgical patients is lower than that of reported ASA series—requiring ICU surveillance for ≥2 days [11]. Scanty literature has focused on outcomes of percutaneous ablation at ages beyond 55 [23] and 60 [24], both pointing at a disproportionate risk of AV block (13–17%). Jahnlová et al. reported the outcomes of ASA on 156 patients beyond 60-years of age [25]. These highly experienced operators reported mortality at 30 days at 2.6%, permanent AV blockage requiring pacemaker at 11.5%, residual significant obstruction was 24%, need for redo septal reduction therapy of 5.2% and 19% remained in advanced heart failure. This data challenges referrals to ASA on the sole basis of age.

The initial SM operation is evolving into a more comprehensive LVOT intervention as is reflected in our analysis. The result of an intervention upon different structures involved in dynamic obstruction at the LVOT may explain consistent obstruction relief. Surgery has the advantage of assessing under direct vision the anatomy contributing to the complex mitral-septal interaction. The concept of an interventional approach limited to the septum, for all, seems contested given the high proportion of abnormalities requiring additional LVOT procedures (65.4%). Division of secondary chordae contributing to SAM has been performed nearly in half of our patients. This contrasts with our recent experience in younger (<65) isolated SM cohort where this has been used less commonly (in one-third of patients). Indeed, a tailored and thoughtful open procedure—beyond influence on the septum alone—planed with the use of imaging tools has been an effective therapeutic option, potentially mitigating the risk of ventricular septal defects in some patients. Frequently, the need for additional concomitant LVOT intervention cannot be anticipated by imaging and requires direct surgical assessment. At times, further resection and LVOT intervention may require a repeat cardiopulmonary bypass run.

Some patients’ anatomy (small LV cavity, rigid mitral leaflets, mitral-LVOT angle) may still preclude a mitral-sparing approach. It could be argued that in HOCM a mitral valve replacement could solve both LVOT obstruction and mitral regurgitation, yielding SM unnecessary. The exclusion of these 3 patients (accounting for 5.4% of our experience) undergoing SM and concomitant mitral replacement could be interpreted as a bias. We believe that patients that could not be treated with septal/LVOT intervention alone constitute a different HCM population. Classically, those patients requiring mitral replacement have been excluded from the majority of SM series. This radical form of HOCM treatment would likely not be offered as a first-line therapeutic option against alcohol septal ablation. It is likely that many of the patients of our cohort would have received a mitral valve replacement if treated in a non-HCM center, which in itself is technically easier. However, we advocate that avoidance of mitral replacement leads to superior hemodynamic gain while obviating the known risks inherent to mitral prostheses (including stroke, bleeding and endocarditis). For the above-mentioned reasons, we elected to selectively assess the outcomes of mitral-sparing SM intervention and belief that this should be the standard of care whenever possible. We aimed at focusing the analysis on elderly patients that are candidates for a mitral sparing approach that should be identified and referred to HCM-referral centers. This data will also facilitate future comparisons to percutaneous septal ablation with or without concomitant structural interventions.

Our study engaged a population with advanced structural disease with the intention to report the safety and efficacy of an operation that is less commonly used [26]. This elderly population may have frequently sustained the consequences of ancient LVOT obstruction. It is reasonable to attribute baseline damage to the left-sided valves and dilatation/stretching of the left atrium to the presence of chronic abnormal flow patterns. Consequently, the need for concomitant atrial fibrillation surgery, aortic valve procedures and the presence of mitral fibro-calcification may arise from uncorrected long-standing pathophysiology. We speculate that earlier interventions could mitigate or prevent such derangements.

With the advent of novel pharmacotherapeutic options, the focus will turn soon to patients’ profiles equivalent to the currently studied. The outcome data reported here for the symptomatic elderly HOCM population sets the bar high for the promising allosteric inhibitor of cardiac myosin ATPase mavacamten [27]. To date, the initial experience with this drug has shown inferior resolution of obstruction and heart failure symptoms—in a less symptomatic and healthier population. Safety concerns on this lifelong pharmacologic treatment cannot be lifted yet on the basis of unpredictable LV function impairment and atrial fibrillation onset. From an efficacy standpoint, 50% of patients persisted with obstruction beyond the threshold for septal reduction intervention. In contrast, 90% of our SM patients achieved complete resolution of obstruction (no provokable gradient at all) and 20% did not require negative inotropic agents at follow-up.

It should be emphasized that the benefits and risks for any given patient should be balanced in expert HCM heart teams.

## 5. Conclusions

Septal myectomy in patients beyond 65 years old is a safe operation and yields excellent outcomes regardless of the frequent need for concomitant procedures. The need for additional LVOT interventions beyond septal myectomy to relieve obstruction is common in this advanced cohort of hypertrophic cardiomyopathy patients. 

## Figures and Tables

**Table 1:** Baseline preoperative characteristics.

Preoperative Characteristics	All Patients (52)
Age	71.5 (65–82)
Female	36 (69.2)
Height (m)	1.6 (1.4–1.9)
Weight (Kg)	72.5 (50–96)
Body mass index (Kg/m^2^)	28 (17.5–40.7)
Hypertension	39 (75)
Atrial fibrillation	13 (25)
Coronary disease	10 (19.2)
COPD	10 (19.2)
Cerebrovascular accident	3 (5.8)
Diabetes mellitus	13 (25)
Creatinine (mg/dL)	0.9 ± 0.3
Ascending aorta calcified atheroma	3 (5.8)
Family sudden cardiac death	3 (5.8)
NYHA III/IV	47 (90.4)
Angina	21 (40.4)
Syncope	6 (11.5)
Preoperative sudden death	1 (1.9)
Sudden cardiac death risk (HCM risk-SCD)	1.8 (0.8–10.4)
EuroSCORE II	3.76 (1–28.7)

Continuous variables are expressed as mean (±standard deviation) or median (range). Qualitative variables are expressed as frequencies (percentage). NYHA: New York Heart Association, COPD: chronic lung disease, HCM: hypertrophic cardiomyopathy, SCD: sudden cardiac death.

**Table 2:** Preoperative morphological and functional data.

Left Ventricular Hypertrophy Patterns	
Basal	34 (65.4)
Long segment	15 (28.8)
Long segment and apical obliteration	1 (1.9)
Midventricular	1 (1.9)
Concentric	1 (1.9)
**Left ventricular ejection fraction (%)**	62 ± 6
**Maximum septal thickness (mm)** **Posterior left ventricular wall thickness (mm)**	26 ± 5 13 ± 2
**Highest intraventricular gradient (mmHg)**	80 (50–220)
**Left atrial diameter (mm)**	43 ± 9
**Mitral valve data**	
Systolic anterior motion (at rest)	44 (84.6)
Systolic anterior motion (with provocation)	52 (100)
Mitral valve prolapse	4 (7.7)
Mitral valve stenosis (< moderate)	3 (5.8)
> Moderate mitral regurgitation	36 (69.2)
Organic mitral regurgitation (beyond SAM)	22 (42.3)
Calcified posterior mitral leaflet	18 (34.6)
Calcified mitral annulus	16 (30.8)
Calcific/fibrotic anterior mitral leaflet	6 (11.5)
**Moderate to severe aortic stenosis**	14 (28.8)
**Moderate to severe aortic regurgitation**	7 (13.4)

Continuous variables are expressed as mean (±standard deviation) or median (range). Qualitative variables are expressed as frequencies (percentage). SAM: systolic anterior motion.

**Table 3:** Intraoperative additional LVOT interventions and concomitant procedures.

**Additional LVOT Procedures (beyond Myectomy)** **for Obstruction Relief**	**34 (65.4)**
Secondary mitral valve chordae division	27 (51.9)
Division of muscular connections to the ventricular septum	12 (23.1)
Division of tendinous connections to the ventricular septum	10 (19.2)
Anterior mitral valve leaflet horizontal plication	4 (7.7)
Papillary muscle repositioning	2 (3.8)
Papillary muscle relief	5 (9.6)
Fibrotic membrane resection	4 (7.7)
Trigonal release	4 (7.7)
**Other cardiac concomitant procedures**	44 (84.6)
Number of concomitant procedures *1* *2* *≥3*	25 (48.1) 8 (15.4) 11 (22.1)
Aortic valve replacement	20 (38.5)
Coronary surgery	8 (15.4)
Maze procedure	10 (19.2)
Mitral valve prolapse repair	4 (7.7)
Aorta surgery	3 (5.8)
Tricuspid valve annuloplasty	2 (3.8)
Other concomitant procedures	12 (23.1)
Cardiopulmonary bypass time (min)	89 (40–166)
Aortic occlusion time (min)	73 (29–129)

Continuous variables are expressed as median (range). LVOT: left ventricular outflow tract.

**Table 4:** Postoperative (in-hospital) data and follow up.

**Postoperative Data**	
ICU length of stay, days	1 (1–8)
**Postoperative complications**	
Mortality (in hospital or at 90 days) *	1 (1.9)
New complete AV block (PPM implantation during hospital stay)	1 (1.9)
Permanent stroke **	1 (1.9)
Iatrogenic ventricular septal defect	0
Reoperation for bleeding	0
Mediastinitis/sternal dehiscence	0
Renal failure requiring temporary dialysis	2 (3.8)
Pneumonia	1 (1.9)
De novo transitory atrial fibrillation	16 (30.8)
**Postprocedural echocardiographic data**	
Mean postoperative gradient	4 ± 14
Complete resolution of obstruction (no residual gradient at all)	46 (88.5)
**Last follow up data**	
NYHA I	42 (82.3)
NYHA II	9 (17.6)
Redo septal reduction therapy (myectomy or ASA)	0
Sudden death or cardiac death	0

Continuous variables are expressed as mean (±standard deviation) or median (range). ICU: intensive care unit, PPM: permanent pacemaker, AV: auriculo-ventricular, NYHA: New York Heart Association * Urgent patient with supra-systemic pulmonary artery hypertension and home oxygen not amenable to other therapies. ** Patient with preoperative bilateral carotid endarterectomy and residual carotid obstruction.

## Data Availability

The data that support the findings of this study is available from the Hospital Clinic of Barcelona database but restrictions apply to the availability of these data, which were used under license for the current study, and so are not publicly available. Data might however be available upon reasonable request through IRB and corresponding author permission.

## Abbreviations and Acronyms

ASA	Alcohol septal ablation
HCM	Hypertrophic cardiomyopathy
HOCM	Hypertrophic obstructive cardiomyopathy
LVOT	Left ventricular outflow tract
NYHA	New York Heart Association
SAM	Systolic anterior motion of the mitral valve
SCD	Sudden cardiac death
SM	Septal myectomy
SRT	Septal reduction therapy
